# Fatal coinfection with Oropouche virus and influenza A(H1N1)pdm09: Case report and post-mortem findings

**DOI:** 10.1016/j.imj.2026.100271

**Published:** 2026-06-27

**Authors:** Lucas Vieira de Lima, Marcos Adriano Garcia Campos, Romullo José Costa Ataides, Kwang Il Marciaga Teofilo, Pedro Manuel Barros de Sousa, Gyl Eanes Barros Silva

**Affiliations:** a University Hospital of the Federal University of Maranhão, Department of Pathology, São Luís 65020-070, Brazil; b Global Emergency Medicine Innovation and Implementation Research Center, Duke University School of Medicine, Durham 27710, USA; c Johns Hopkins Bloomberg School of Public Health, Department of Epidemiology, Baltimore 21205, USA

**Keywords:** Oropouche virus, Influenza A, Coinfection, Autopsy

## Abstract

Oropouche virus (OROV) is an emerging orthobunyavirus that causes increasingly severe and deadly disease in Brazil. However, coinfection with influenza A(H1N1)pdm09 has not been previously described in the published literature, to our knowledge. We report the first documented fatal case of OROV and influenza A(H1N1)pdm09 coinfection in a previously healthy 16-year-old female from rural Maranhão, Brazil. The patient presented with dyspnea, odynophagia, and hemoptysis and deteriorated rapidly, requiring orotracheal intubation on day three of illness due to massive endobronchial hemorrhage, followed by fatal hemodynamic collapse. Autopsy revealed bilateral serosanguineous pleural effusion, diffuse alveolar hemorrhage, pericardial petechiae, acute tubular necrosis, and cerebral edema with tonsillar herniation. Real-time reverse-transcriptase polymerase chain reaction confirmed OROV in lung, liver, spleen, and lymph node tissues, and influenza A(H1N1)pdm09 in both nasal swab and lung tissue; all other arboviruses and respiratory pathogens tested negative. The histopathological findings provide, to our knowledge, the first histopathological characterization of OROV-associated microvascular injury and suggest synergistic thromboinflammatory pathways with influenza A.

This case broadens the recognized clinical spectrum of OROV and highlights the need for systematic coinfection screening and coagulation monitoring in severe arboviral presentations, even in young patients without comorbidities.

## Highlights

• First reported fatal OROV and influenza A(H1N1)pdm09 coinfection.
• Severe disease occurred in a previously healthy 16-year-old girl.
• Massive hemoptysis marked the rapid and fatal clinical deterioration.
• Autopsy revealed diffuse alveolar hemorrhage and pulmonary edema.
• Findings broaden the spectrum of severe and atypical OROV disease.

## 1 Introduction

Oropouche virus (OROV) is one of the most important emerging diseases in Brazil. It is a potential candidate to cause the next epidemic in the Americas, alongside Venezuelan equine encephalitis and Mayaro fever.^1^ The most common clinical presentation includes high fever, myalgia, arthralgia, nausea, vomiting, and photophobia.^2^ Less frequently, meningitis, encephalitis, and hemorrhagic phenomena, such as epistaxis, petechiae, and gingival bleeding, may occur.^2^ Transmission occurs primarily via bites of infected biting midges (mainly *Culicoides paraensis*), with an incubation period of 3–8 days.^3^ Fatal cases of the disease in adult individuals have been recently reported, as well as fetal deaths and cases of microcephaly in neonates related to the OROV, suggesting a potential for vertical transmission.^4^

In contrast, influenza A[hemagglutinin 1, neuraminidase 1(H1N1)]pdm09 is an established global concern, causing annual epidemics and sporadic pandemics.^5^ In April 2009, the World Health Organization declared a new international public health emergency caused by the novel H1N1 virus, which is transmitted through direct contact or respiratory secretions from infected individuals.^6^ Most cases affected children and young adults, with a low lethality rate (< 1%), typically presenting with mild to moderate flu-like symptoms.^7^ Fatalities were more common in patients with chronic comorbidities.^7^

There are few reports on the clinical impact of coinfection between non-respiratory viruses and H1N1,^8^ and to our knowledge, no study has described coinfection with OROV. Previous coinfections reported in OROV cases included *Leptospira* and dengue virus serotype 2 in non-fatal instances.^9^ The following report describes an atypical case involving an adolescent with coinfection of OROV and H1N1 with fatal outcome, and its post-mortem findings.

## 2 Case presentation

A 16-year-old female with no previous comorbidities presented with dyspnea, odynophagia, and hemoptysis-associated cough. She was from a rural area in Maranhão, a state of autochthonous OROV transmission located near the Brazilian Amazon region, and denied recent travel. She sought emergency care, two days after onset of symptoms, where a chest X-ray suggested pneumonia, resulting in her hospitalization. Her condition progressively worsened on the next day, with increased dyspnea and cough accompanied by headache and right-sided chest pain. Orotracheal intubation was performed on the third day of illness, when massive bleeding through the endotracheal tube was identified. The patient had a hemodynamic worsening, and subsequently died.

**Fig. 1:**
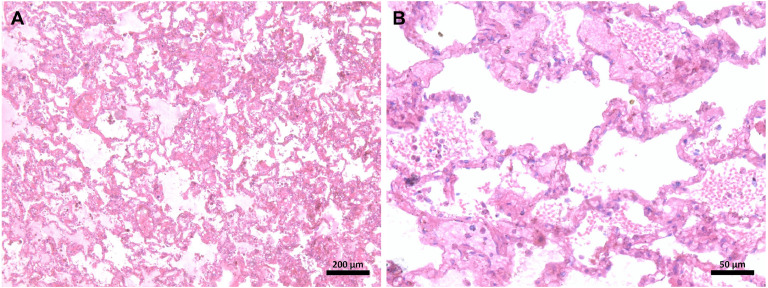
Pulmonary histopathological findings in a fatal case of Oropouche virus and influenza A(H1N1)pdm09 confection. Hematoxylin and eosin (H&E) staining of lung tissue sections. (A) Low-power view showing alveolar spaces diffusely filled with erythrocytes and macrophages, consistent with diffuse alveolar hemorrhage and pulmonary congestion (scale bar = 200 µm). (B) High-power view demonstrating siderophages (hemosiderin-laden macrophages) within alveolar spaces, with thickened alveolar septa and vascular congestion (scale bar = 50 µm).

The autopsy revealed a bilateral large serosanguineous pleural effusion totaling approximately one liter. The lungs exhibited a purplish color with hemorrhagic spots; cut surfaces showed congestion and hemorrhage; petechiae were diffusely distributed in the pericardium, along with mild cerebral edema and tonsillar herniation. Microscopic findings showed diffuse alveolar hemorrhage and pulmonary edema (Fig. 1). The kidneys showed acute tubular necrosis with vascular congestion. The liver exhibited only vascular congestion.

Samples were collected for reverse transcriptase polymerase chain reaction testing for arboviruses and respiratory viruses. Results were positive for OROV and H1N1. For the complete list of infectious agents tested, see Table 1. For the other infectious agents, the results were negative.

**Table 1:** List of infectious agents tested.

Agent	Method	Material	Result
Oropouche	Real-time RT-PCR	Tissue (lung, liver, spleen, lymph node)	Positive
Influenza	Real-time RT-PCR	Nasal swab	Positive
Influenza	Sequencing (genome)	Nasal swab	Influenza A(H1N1)pdm09-like
Influenza	Real-time RT-PCR	Tissue (lung)	Positive, influenza A(H1N1)pdm09
COVID-19	Real-time RT-PCR	Nasal swab	Negative
Leptospirosis	Real-time RT-PCR	Tissue (lung, liver)	Negative
Dengue	Real-time RT-PCR	Tissue (spleen, lymph node)	Negative
Mayaro	Real-time RT-PCR	Tissue (liver, spleen, lung, lymph node).	Negative
Chikungunya	Real-time RT-PCR	Tissue (spleen)	Negative
Zika	Real-time RT-PCR	Tissue (spleen)	Negative
Hantavirus	Real-time RT-PCR	Tissue (liver, spleen, lung, lymph node, brain).	Negative

*Abbreviations*: COVID-19, coronavirus disease 2019; RT-PCR, reverse transcriptase polymerase chain reaction.

## 3 Discussion

We describe a rare case of fatal OROV with H1N1 coinfection. The patient had no serious pre-existing illnesses, and symptoms progressed rapidly and severely. However, OROV shares symptoms with other arboviral diseases,^9^ and reports of fatal OROV present with acute hemorrhagic manifestations that rapidly lead to death.^10^

Hemorrhagic manifestations related to OROV have already been described in a series of cases in 2007–2008 in the Brazilian Amazon Region, in which 15.5% (20 patients) presented hemorrhagic phenomena (petechiae, epistaxis, and gingival bleeding), and all patients recovered without complications.^11^ Laboratory abnormalities related to severe bleeding were confirmed in two fatal cases in patients under 25 years of age without comorbidities, from a nonendemic region in Brazil, in which, approximately 4 days after symptom onset, a rapid decline in hematocrit, thrombocytopenia, and prolongation of clotting time were observed, as well as elevated liver enzymes and renal dysfunction.^10^ Symptoms reported included bronchial hemorrhage, severe abdominal pain, epistaxis, vaginal bleeding, and cold and clammy skin, in addition to widespread petechiae.^10^

And now, in our case, we describe alterations verified at the histopathological level, confirming the severe pattern of hemorrhage in pulmonary tissue, in a young patient without comorbidities, with an unfavorable outcome within a few days of the onset of symptoms. In a mouse model, OROV was found to spread rapidly through the central nervous system, crossing the blood-brain barrier, which may explain severe cases of encephalitis.^12^ Another study found that OROV was detectable in the liver 6 hours after intracerebral inoculation in 3-week-old hamsters, indicating hematogenous spread from the brain to the liver, with associated lesions and marked hepatocyte necrosis.^13^

Although the pathophysiology of severe, rapidly progressing OROV cases remains unclear, lipoprotein receptor–related protein one has been identified as a key host factor for viral entry, as seen in Rift Valley fever. This bunyavirus infection can cause hemorrhagic syndrome, underscoring its broader importance in the same virus family.^14^ This cellular entry mechanism, combined with the thromboinflammatory response typical of severe H1N1,^15^ likely converged to cause the fatal outcome.

These effects could have been worsened by coinfection with H1N1. As an example, an experimental study in mice investigated the impact of coinfection with a neurotropic RNA virus (Semliki Forest virus) and influenza A virus (IAV).^16^ It was observed that infection with Semliki Forest virus about eight days before IAV infection resulted in prolonged IAV replication, increased cytokine and chemokine levels, exacerbated pulmonary responses, inefficient CD8^+^ T-cell activation, and dendritic cell paralysis. Additionally, increased blood-brain barrier permeability, presence of IAV RNA in brain tissue, and an exacerbated response to IAV were noted.^16^ Coinfection mechanism between OROV and H1N1 are scarce in the literature.

On a systemic level, OROV triggers thromboinflammation, a dangerous cycle where blood clotting and extreme inflammation occur simultaneously.^17^ This process can lead to endothelial damage (injury to blood vessel linings) and is responsible for the hemorrhagic symptoms seen in about 15% of patients.^17^ OROV may also reach the brain by hiding inside white blood cells that bypasses the blood-brain barrier and sparks a toxic inflammatory response.^18^ These mechanisms are very similar to those found in other viruses like dengue and chikungunya, where a “cytokine storm” and vascular dysfunction drive the severity of the disease.^15^ Furthermore, the finding of cerebral edema and tonsillar herniation indicates a terminal increase in intracranial pressure, likely precipitated by severe hypoxia and systemic vascular leak. This neurological catastrophe, rather than isolated cardiac pathology, explains the progression to death. The pericardial punctate hemorrhages further support a state of widespread microvascular fragility, suggesting that the OROV and H1N1 coinfection acted synergistically to induce a fatal systemic vascular breakdown.

A significant limitation of this study is the inherent nature of case reports, which preclude establishing definitive causal links between coinfection and clinical severity. While we observed fatal hemorrhagic manifestations, it remains unclear whether these were exclusively driven by OROV, influenza A(H1N1)pdm09, or a synergistic interaction between the two. Specifically, the observed coagulopathy could have been an independent manifestation of severe influenza, which is known to induce systemic vascular dysregulation and thromboinflammation.^15^ Consequently, while our findings highlight a potential broadening of the OROV clinical spectrum, they should be interpreted as a hypothesis-generating observation rather than a confirmed expansion of the virus’s standalone pathology.

## 4 Conclusions

New epidemics of emerging or re-emerging infectious diseases with greater infectivity and transmission potential are increasingly common, causing substantial morbidity and mortality, often due to pathogenic, environmental, and population factors related to climate change. That can expand the reservoirs of potential vectors for many diseases, increase pathogen-host interactions, and degrade the overall health of susceptible human populations, leading to new epidemic outbreaks.

Severe cases of OROV in nonendemic areas have become increasingly common. Symptoms of dengue, Zika, and chikungunya are similar to OROV. They can lead to confusion in clinical diagnosis, especially in cases of suspected coinfection, requiring specific investigation by the health team and strengthened epidemiological surveillance.

Although most studies describe mild symptoms related to OROV, we contribute to the literature with another atypically severe case, with an unreported H1N1 coinfection. Similar to other reports, a young patient without comorbidities was affected, with death occurring in less than a week from the onset of symptoms, highlighting the hemorrhagic manifestations as a warning sign, and the need for research into coagulation disorders in these patients. The histopathological findings presented may be key to determining the extent and severity of OROV and H1N1 involvement in the microvasculature.

## CRediT authorship contribution statement

**Lucas Vieira de Lima:** Writing – original draft, Data curation, Conceptualization. **Marcos Adriano Garcia Campos:** Writing – original draft, Methodology. **Romullo José Costa Ataides:** Writing – review & editing. **Kwang Il Marciaga Teofilo:** Investigation, Data curation. **Pedro Manuel Barros de Sousa:** Investigation, Data curation. **Gyl Eanes Barros Silva:** Writing – review & editing, Supervision.

## Informed consent

Written informed consent was obtained from the patient’s parents for conduct the research, publication of this manuscript and any accompanying images.

## Organ donation

No organs were donated or transplanted in this case. Autopsy was performed for diagnostic purposes only.

## Ethical statement

The study was approved by the Research Ethics Committee of the University Hospital of the Federal University of Maranhão (HU/UFMA), under code number 4.750.825.

## Data availability

The data that support the findings of this study are available from the corresponding author upon reasonable request.

## Animal treatment

Not applicable.

## Generative AI

The authors used generative artificial intelligence tools to assist with language editing and manuscript preparation. All scientific content, data, interpretations, and conclusions were generated and verified by the authors, who take full responsibility for the integrity of the work.

## Funding

This research did not receive any specific grant from funding agencies in the public, commercial, or not-for-profit sectors.

## Declaration of competing interest

The authors declare that they have no known competing financial interests or personal relationships that could have appeared to influence the work reported in this paper.

## Appendix Supplementary materials

Image, application 1
