# Supplementary material for: Fatal coinfection with Oropouche virus and influenza A(H1N1)pdm09: Case report and post-mortem findings
Source: Infect Med (Beijing). 2026 Jun 27;5(3):100271. doi: 10.1016/j.imj.2026.100271 (PMC13382114; doi:10.1016/j.imj.2026.100271)
Supplement: Supplementary file 1 [file mmc1.docx]

**Additional References**

[11.](https://www.zotero.org/google-docs/?e4dFei) [Mourão MPG, Bastos MS, Gimaque JBL, Mota BR, Souza GS, Grimmer GHN, et al. Oropouche Fever Outbreak, Manaus, Brazil, 2007–2008. Emerg Infect Dis. 2009 Dec;15(12):2063–4.](https://www.zotero.org/google-docs/?e4dFei)

[12.](https://www.zotero.org/google-docs/?e4dFei) [Santos RI, Bueno-Júnior LS, Ruggiero RN, Almeida MF, Silva ML, Paula FE, et al. Spread of Oropouche virus into the central nervous system in mouse. Viruses. 2014 Oct 10;6(10):3827–36.](https://www.zotero.org/google-docs/?e4dFei)

[13.](https://www.zotero.org/google-docs/?e4dFei) [Araújo R, Dias LB, Araújo MT, Pinheiro F, Oliva OF. [Ultrastructural changes in the hamster liver after experimental inoculation with Oropouche arbovirus (type BeAn 19991)]. Rev Inst Med Trop Sao Paulo. 1978;20(1):45–54.](https://www.zotero.org/google-docs/?e4dFei)

[14.](https://www.zotero.org/google-docs/?e4dFei) [Wesselmann KM, Postigo-Hidalgo I, Pezzi L, de Oliveira-Filho EF, Fischer C, de Lamballerie X, et al. Emergence of Oropouche fever in Latin America: a narrative review. Lancet Infect Dis. 2024 July;24(7):e439–52.](https://www.zotero.org/google-docs/?e4dFei)

15. Batista VL, Martins JR, Queiroz-Junior CM, Hottz ED, Teixeira MM, Costa VV. Mechanisms of Thromboinflammation in Viral Infections-A Narrative Review. Viruses. 2025 Sep 3;17(9):1207.

[16.](https://www.zotero.org/google-docs/?e4dFei) [Foo IJH, Chua BY, Clemens EB, Chang SY, Jia X, McQuilten HA, et al. Prior infection with unrelated neurotropic virus exacerbates influenza disease and impairs lung T cell responses. Nat Commun. 2024 Mar 23;15(1):2619.](https://www.zotero.org/google-docs/?e4dFei)

17. Morley D, Kennedy E, Dowall S. Preclinical Models of Oropouche Virus Infection and Disease. Pathogens. 2025 Dec 11;14(12):1272.

18. Porwal S, Malviya R, Sridhar SB, Shareef J, Wadhwa T. Mysterious Oropouche virus: Transmission, symptoms, and control. Infect Med (Beijing). 2025 Mar 17;4(2):100177.
